# The Landscape of Immune Cells Indicates Prognosis and Applicability of Checkpoint Therapy in Hepatocellular Carcinoma

**DOI:** 10.3389/fonc.2021.744951

**Published:** 2021-09-28

**Authors:** Jiacheng Huang, Lele Zhang, Jianxiang Chen, Dalong Wan, Lin Zhou, Shusen Zheng, Yiting Qiao

**Affiliations:** ^1^ Division of Hepatobiliary and Pancreatic Surgery, Department of Surgery, The First Affiliated Hospital, Zhejiang University School of Medicine, Hangzhou, China; ^2^ School of Medicine, Zhejiang University, Hangzhou, China; ^3^ National Health Center (NHC) Key Laboratory of Combined Multi-organ Transplantation, Hangzhou, China; ^4^ Key Laboratory of the Diagnosis and Treatment of Organ Transplantation, Research Unit of Collaborative Diagnosis and Treatment for Hepatobiliary and Pancreatic Cancer, Chinese Academy of Medical Sciences (2019RU019), Hangzhou, China; ^5^ Key Laboratory of Organ Transplantation, Research Center for Diagnosis and Treatment of Hepatobiliary Diseases, Hangzhou, China; ^6^ Shulan (Hangzhou) Hospital Affiliated to Zhejiang Shuren University Shulan International Medical College, Hangzhou, China; ^7^ Pharmacy Institute and Department of Hepatology, Institute of Hepatology and Metabolic Diseases, Institute of Integrated Chinese and Western Medicine for Oncology, the Affiliated Hospital of Hangzhou Normal University, Hangzhou, China; ^8^ Key Laboratory of Elemene Class Anti-Cancer Medicine of Zhejiang Province, Hangzhou, China; ^9^ Engineering Laboratory of Development and Application of Chinese Medicine from Zhejiang Province, Hangzhou, China; ^10^ Collaborative Innovation Center of Chinese Medicines from Zhejiang Province, School of Medicine, Hangzhou Normal University, Hangzhou, China

**Keywords:** HCC, CIBERSORT, PD-L1, immune subtype, prognosis

## Abstract

**Background:**

Tumor-infiltrating immune cells are important components of tumor microenvironment (TME), and their composition reflects the confrontation between host immune system and tumor cells. However, the relationship between the composition of infiltrating immune cells, prognosis, and the applicability of anti-PD-1/PD-L1 therapy in hepatocellular carcinoma (HCC) needs systematic examination.

**Methods:**

Cell-Type Identification by Estimating Relative Subsets of RNA Transcripts (CIBERSORT) was applied to evaluate the infiltration of immune cells based on The Cancer Genome Atlas (TCGA) liver hepatocellular carcinoma (LIHC) cohort. Diagnostic and prognostic models were constructed based on immune cells, and the models were validated by two external cohorts. The relationship between immune cells and PD-L1 was evaluated by Spearman correlation, and the finding was validated in our in-house HCC sample.

**Result:**

Patients in TCGA LIHC cohort were classified into six subtypes with different prognosis based on the proportion of tumor-infiltrating immune cells simulated *via* CIBERSORT. Among 22 types of immune cells, intratumoral PD-L1 mRNA level exhibited linear relationship with the fraction of five types of immune cells (M1 macrophages, plasma cells, CD8^+^ T cells, resting mast cells, and regulatory T cells), and M1 macrophages showed the strongest relevance (*R* = 0.26, *p* < 0.001). Immunohistochemistry of our in-house HCC specimens verified this conclusion. Moreover, intratumoral mRNA levels of M1 macrophage-associated cytokines were positively correlated with PD-L1 level.

**Conclusions:**

Our study demonstrated that the prognosis of HCC patients was associated with the pattern of infiltrating immune cells in TME, and macrophage-associated cytokines might be a potential non-invasive marker for predicting the PD-L1 level for HCC patients.

## Background

Hepatocellular carcinoma (HCC) is a heavy burden for the healthcare system worldwide. According to global cancer statistics in 2018, HCC ranked fifth in newly diagnosed cancer cases and fourth in mortality (1). The lack of hallmark symptoms impairs the efficacy of early detection, so patients are often diagnosed with advanced HCC at the time of initial diagnosis. Surgery is inapplicable to them in most circumstances, and palliative locoregional therapies (transcatheter arterial chemoembolization, percutaneous ethanol injection, and radiofrequency ablation) can only control the disease progression for a short period (2). The multikinase inhibitor Sorafenib is offered to advanced HCC patients as the standard first-line therapy, but its objective response rate as a monotherapy is dismal, ranging from 2% to 3% (3). Generally, the treatment of advanced HCC remains highly challenging, and the 5-year survival rate of these patients is only 5–6% (4).

Cancer is a chronic disease involving constant interaction with host immune system. Immune therapy is a new category of cancer therapy, which is aimed to reactivate host antitumor immunity either by interrupting the immune checkpoint complexes hijacked by tumor cells [e.g., blocking antibodies for programmed cell death protein 1/programmed death ligand 1 (PD-1/PD-L1) and cytotoxic T-lymphocyte-associated protein 4 (CTLA-4)] or by infusing genetically engineered immune cells [e.g., chimeric antigen receptor (CAR)-T-cell therapy]. Recently, nivolumab (PD-1 monoclonal antibody) has been approved for HCC treatment, and its objective response rate reached 15%–20% (5). In addition, the objective response rate increased from 17% to 27% when PD-L1 monoclonal antibody atezolizumab was applied in combination with vascular endothelial growth factor (VEGF) monoclonal antibody bevacizumab in advanced HCC, compared to the monotherapy of atezolizumab (6). Therefore, immune therapy mediated by blocking antibodies for PD-1/PD-L1 is a promising therapeutic strategy for advanced HCC. However, patients vary dramatically in their sensitivity to checkpoint therapy, so a methodology to accurately predict their prognosis and drug sensitivity is urgently needed to establish a drug guidance system for its application in HCC.

Several studies implied that patients’ responses to blocking antibodies for PD-1/PD-L1 were strongly associated with both the intratumoral level of PD-L1 and the status of their tumor microenvironment (TME) (7, 8). Both basic research and translational studies have demonstrated that many components of TME could induce immunosuppression to resist attacks from host immune system.

In HCC, tumor cells, infiltrating lymphocytes, and other components of TME all contribute to the maintenance of immunosuppression. For example, the number of CD4^+^ T helper cells and CD8^+^ cytotoxic T cells decreased (9), while immunosuppressive cells such as T regulatory cells (10) and invariant natural killer T cells (iNKT) (11) increased in HCC. M1 macrophages with an antitumor capacity transform into M2 macrophages in favor of HCC progression (12). Moreover, less costimulatory signals such as CD80/CD86 are present on the membrane of antigen-presenting cells (13), while more inhibitory checkpoint signals such as CTLA-4 and PD-L1 are abnormally expressed on the surface of HCC cells. Therefore, a comprehensive understanding of the interplay among cells within TME, especially immune cells, is one of the fundamental steps to establish a drug guidance system for anti-PD-1/PD-L1 therapies in HCC.

In our routine hematoxylin–eosin (H&E) staining analysis on HCC specimens, we noticed that considerable areas of tumor tissues were occupied by infiltrated immune cells (**Supplementary Figure S1**). Transcriptome based on the second-generation sequencing of bulk tissue actually contains the transcriptional information of both tumor cells and infiltrated immune cells. However, it requires specialized bioinformatic tools to extract the information of immune cells from the total transcriptome. In recent years, many deconvolution algorithms have been developed to quantitatively evaluate the type and number of infiltrated immune cells on the basis of the transcriptome of bulk tumor. Cell-type Identification by Estimating Relative Subsets of RNA Transcripts (CIBERSORT), a web-based deconvolution algorithm, provides the estimation of the relative fraction of different immune cells based on gene expression (14). Compared to single-cell sequencing method, deconvolution algorithms have the advantages of prices, throughputs, and compatibility with frozen specimens. Therefore, these methods are frequently applied in retrospective studies for cancer immunology (15).

Our present study applied CIBERSORT algorithm to analyze the landscape of immune cell infiltration based on The Cancer Genome Atlas (TCGA) liver hepatocellular carcinoma (LIHC) cohort and constructed both diagnostic and prognostic models. Two independent cohorts, GSE76427 and Singapore cohort, were applied to validate the model. The relationship between infiltrated immune cells and intratumoral PD-L1 mRNA level was analyzed. Moreover, immunohistochemical staining was performed on HCC specimens from our center for the verification of bioinformatic discoveries, and the relationship between the intratumoral mRNA levels of M1 macrophage-associated cytokines and PD-L1 was explored.

## Methods

### Acquisition of TCGA-LIHC Cohort

RNA-seq data of LIHC cohort was downloaded from The Cancer Genome Atlas (TCGA: https://portal.gdc.cancer.gov/). High-throughput sequencing counts (HTSeq counts) value was applied for further analysis. The ENSEMBLE name of genes was converted to SYMBOL according to the annotation file “Homo_sapiens.GRCh38.100.gtf” downloaded from Ensembl genome browser 100 (http://asia.ensembl.org/index.html). In addition, the clinical information including prognostic data was also recorded. “TCGAbiolinks” package (16) was utilized for the acquisition of TCGA data (access date: June 24, 2020).

### HCC Sample Collection and Microarray Scanning

Ten normal hepatocellular tissues, 41 para-cancerous tissues, and 76 cancerous tissues were collected from patients who underwent partial hepatectomy in National Cancer Centre Singapore. Total RNA was extracted and hybridized to Affymetrix Human Genome U133 Plus 2.0 Array. More details were previously described (17) (GSE121248). The clinicopathological details are cited in **Table 1**.

**Table 1:** Clinical features of the Singapore HCC cohort.

Characteristic	Variable	n
Age	≥60	41
	<60	35
Gender	Male	66
	Female	10
Tumor venous infiltration	Yes	28
	No	48
Cirrhosis status	Yes	41
	No	35
Tumor size	≤3cm	23
	3–5cm	21
	>5cm	32
AJCC stage	I	46
	II	21
	IIIA	9
Child’s grade	A	56
	B	20

### Estimation of Immune Cells Infiltration

The gene expression matrix was uploaded to CIBERSORT as mixture file and run with 1,000 permutations applying the “LM22” signature. The fraction of 22 types of immune cells sums up to one for each sample. Therefore, it is feasible to compare the fraction of immune cells in different samples.

### The Comparison of Immune Cell Fraction Between Cancerous and Para-Cancerous Tissues

We randomly divided the TCGA-LIHC cohort (375 cancerous samples and 50 para-cancerous samples) into training cohort and test cohort (2:1) when applying “createDataPartition” function in “caret” package in R. Least absolute shrinkage and selection operator (LASSO) is a kind of dimensionality reduction statistical method that constructs penalty function so that the coefficients of some variates are compressed to zero. LASSO-logistic analysis was applied to identify the types of immune cells with significantly different fractions between cancerous and para-cancerous tissues. The diagnostic immune risk score (dIRS) was calculated based on the LASSO-logistic model above, and the accuracy of the model was evaluated by receiver operating characteristic (ROC) curves. After validation with the internal test cohort from TCGA, two independent cohorts (Singapore and GSE76427) were used as external validation for the LASSO-logistic model.

### Prognostic Model of HCC Based on the Fraction of Immune Cells

The endpoint in this prognostic prediction model was overall survival (OS), namely, the endurance from the date of diagnosis to the date of death. Samples recorded from TCGA clinical information were matched to the samples from TCGA RNA-seq data. After removal of the records whose OS was zero, the rest of the tumor samples in TCGA-LIHC cohort were randomly divided into training cohort and test cohort (7:3) when applying “sample” function in R. LASSO-COX regression model was applied to screen for optimal kinds of immune cells, which suggested the prognosis in HCC, and prognostic immune risk score (pIRS) was calculated based on the LASSO-COX model. Similarly, after validation by the internal test cohort from TCGA, an independent cohort (GSE76427) was used as external validation for the LASSO-COX regression model.

### Identification of Immune Subtypes of HCC

To probe the immune subtypes of HCC and the relationship between immune subtypes and prognostic value, we applied hierarchical clustering of immune cells. The survival analysis was performed based on different immune subtypes in HCC.

### Statistical Analysis

All statistical analysis was completed in R version 4.0.0. LASSO analysis was performed by “glmnet” package. “pROC” package was applied to draw ROC curves and calculate the area under the curve (AUC). “survivalROC” package was applied to perform survival analysis and plot the survival curves. Log-rank *p*-value was calculated. Hierarchical clustering of immune cells was carried out by applying “cluster” package. Spearman’s rank correlation coefficient was calculated to appraise the relationship between the fraction of immune cells and the expression of CD274 (PD-L1) mRNA, and relationship between CD274 (PD-L1) expression and the mRNA level of M1 macrophage associated cytokines was also evaluated. Analysis of variance (ANOVA) was applied to compare the PD-L1 level in HCC with different immune subtypes. Chi-square test was applied to evaluate of CD138, CD86, and PD-L1 based on the immunohistochemistry score. *p* < 0.05 was considered statistically significant.

### Gene Set Enrichment Analysis

Gene set enrichment analysis (GSEA) is an algorithm that figures out if preset gene sets display statistically significant differences between two different phenotypes or groups (18). “clusterProfiler” package (19) was applied to perform GSEA analysis.

### Immunohistochemical Staining, Scanning, and Evaluation

Sixty-eight samples were obtained from patients who suffered from hepatocellular carcinoma and underwent hepatectomy in The First Affiliated Hospital, Zhejiang University School of Medicine. Paraffin-embedded tissue was sliced and dewaxed. After antigen retrieval, primary antibodies were incubated with slides at 4°C overnight. Herein, CD138 (rabbit monoclonal to Syndecan-1/CD138, Cat. No. ab128936, Abcam, United Kingdom), CD86 (rabbit polyclonal to CD86, Cat No. DF6332, Affinity Biosciences, USA), and PD-L1 (mouse monoclonal to PD-L1/CD274, Cat No. 66248-1-Ig, Proteintech, USA) were used. After washing, secondary antibodies were incubated at 37°C for 30 min and washed. Then diaminobenzidine (DAB) was applied for color development. Lastly, all sections were scanned by Pannoramic DESK, P-MIDI, P250, P1000 (3D HISTECH; Hungary) and were read by Pannoramic Scanner (3D HISTECH; Hungary).

All sections were evaluated by three pathologists independently. CD138 and CD86 were categorized into positive (+) or negative (−), and PD-L1 was scored ranging from 0 to 3 (0, 1, 2, and 3). If there was disagreement of the evaluation, the results of CD138 and CD86 adhered the principal of minority obeying majority, and the final score of PD-L1 was the average of values from three observers. The score of PD-L1 was categorized into two groups (high and low) according to the median value.

## Results

### Stratification of HCC Patients With Different Prognosis Based on Immune Cells Infiltration Landscape

**Figure 1:**
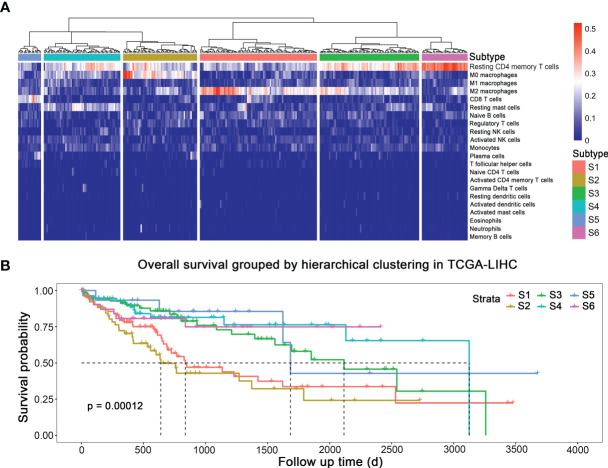
Subtypes of HCC with different prognosis based on immune cells infiltration. **(A)** Immune subtypes. **(B)** Survival curves of different subtypes.

**Figure 1A** displays the fraction of 22 immune cells infiltration simulated by CIBERSORT algorithm. To probe different subtypes of HCC according to their patterns of immune cells infiltration, we constructed a clustering tree (**Figure 1A**), and six subtypes were identified, named as S1, S2, S3, S4, S5, and S6. The numbers of patients belonging to these six subtypes were 87, 55, 74, 57, 17, and 34, respectively. Their survival curves indicated that the prognosis of these six subtypes of HCC was different statistically (**Figure 1B**; *p* < 0.001). The median survival time of S1, S2, S3, S4, and S5 was 837, 639, 2,116, 3,125, and 1,685 days, respectively. S6 did not reach 50% survival by the end of analysis. S2, the subtype with shortest median survival time, was characterized by a high fraction of M0 (unpolarized) macrophage. S1 had a high proportion of M2 macrophage infiltration, and its prognosis was also short. S5 had the best prognosis, and its proportion of CD8^+^ T cells was high. S6 possessed the highest infiltration of resting CD4^+^ memory T cells, followed by S3, and S4 had the moderate proportion of resting CD 4^+^ memory T cells and resting mast cells. Therefore, immune subtypes based on hierarchical clustering could indicate the prognosis in HCC.

### Model for the Discrimination Between Cancerous and Para-Cancerous Tissues

**Figure 2:**
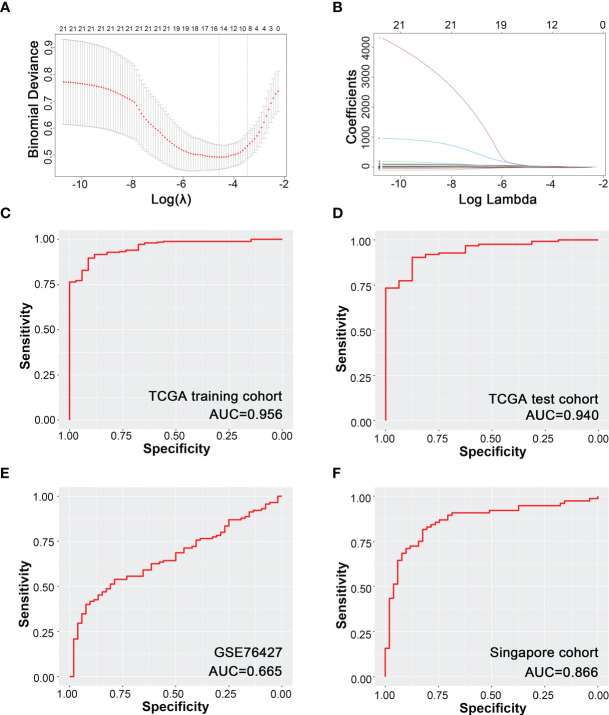
Diagnostic model of HCC based on immune cells infiltration. **(A)** Binominal deviance for different numbers of variables simulated by LASSO-logistic regression model. **(B)** Coefficients of different numbers of variables in LASSO regression model. **(C)** Modeling based on TCGA training cohort. **(D)** Internal test based on TCGA test cohort. **(E)** External validation based on GSE76427. **(F)** External validation based on Singapore cohort.

Ten promising indicators in TCGA training cohort were screened out with non-zero coefficients in our LASSO-logistic model (**Figures 2A, B** **)**. Our LASSO-logistic model was listed as follows: diagnostic immune risk score (dIRS) = 3.2218 − 3.0842 × fraction level of plasma cells + 3.5269 × fraction level of T follicular helper cells + 14.8776 × fraction level of regulatory T cells − 0.3456 × fraction level of resting NK cells − 15.4541 × fraction level of monocytes + 0.4739 × fraction level of M0 macrophages − 3.1345 × fraction level of M2 macrophages + 0.7424 × fraction level of resting mast cells − 25.7202 × fraction level of activated mast cells − 0.0594 × fraction level of neutrophils. The area under the curves (AUCs) in receiver operating characteristic (ROC) curves were 0.956, 0.940, 0.665, and 0.866 in TCGA training cohort (**Figure 2C**), TCGA test cohort (**Figure 2D**), GSE76427 (**Figure 2E**), and Singapore cohort (**Figure 2F**), respectively. Therefore, the model based on immune cells could discriminate cancer and para-cancerous tissues.

### Prognostic Model Based on the Fraction of Immune Cells

**Figure 3:**
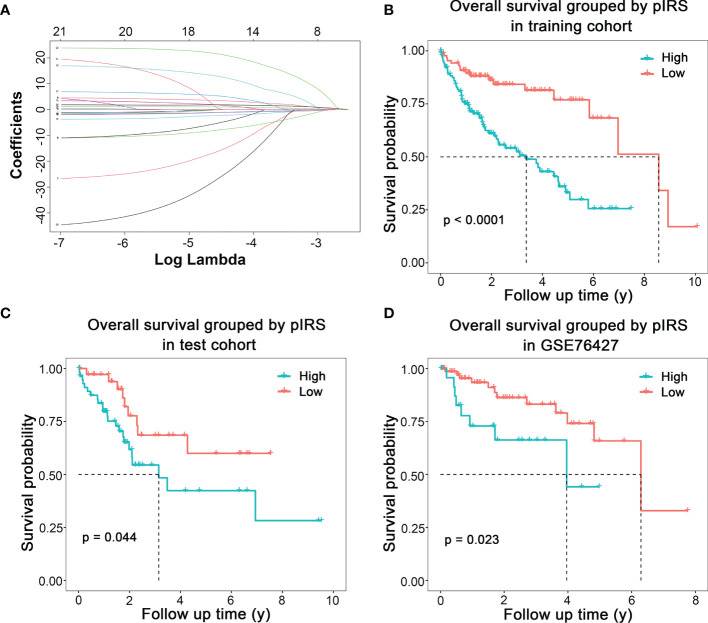
Prognostic model of HCC based on immune cells infiltration. **(A)** Coefficients of different numbers of variables in LASSO regression model. **(B)** Modeling based on TCGA training cohort. **(C)** Internal test based on TCGA test cohort. **(D)** External validation based on GSE76427.

Fourteen variables were selected for the construction of prognostic model (**Figure 3A**). Prognostic immune risk score (pIRS) was calculated as follows: pIRS = −14.3689 × fraction level of memory B cells − 7.4510 × fraction level of plasma cells − 0.9996 × fraction level of resting CD4^+^ memory T cells − 4.3028 × fraction level of T follicular helper cells + 3.3252 × fraction level of regulatory T cells − 2.1403 × fraction level of activated NK cells + 1.4536 × fraction level of monocytes + 1.7866 × fraction level of M0 macrophages + 0.9861 × fraction level of M2 macrophages + 4.8421 × fraction level of resting dendritic cells + 12.0263 × fraction level of activated dendritic cells − 1.0604 × fraction level of resting mast cells − 23.8541 × fraction level of activated mast cells + 20.7487 × fraction level of neutrophils. The survival curves showed that high pIRS indicated poor prognosis in TCGA training cohort (**Figure 3B**, *p* < 0.001), TCGA test cohort (**Figure 3C**, *p* = 0.044), and GSE76427 (**Figure 3D**, *p* = 0.023). Therefore, the model based on immune cells could indicate the prognosis of HCC patients.

### Potential Pathways Related to High pIRS Scores

**Figure 4:**
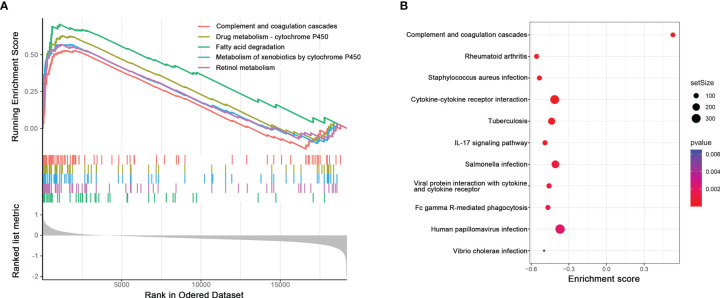
**(A)** Potential pathways related to high pIRS scores based on GSEA. **(B)** Immune-related pathways among GSEA KEGG analysis.

Top 5 Kyoto Encyclopedia of Genes and Genomes (KEGG) pathways related to high pIRS scores, including fatty acid degradation, retinol metabolism, metabolism of xenobiotics by cytochrome P450, drug metabolism–cytochrome P450, and complement and coagulation cascades, are plotted in **Figure 4A**. Additionally, the complete results of GSEA KEGG analysis are listed in **Supplementary Table S1**. Meanwhile, we also found several immune-related pathways among them (**Figure 4B**), namely, complement and coagulation cascades, rheumatoid arthritis, *Staphylococcus aureus* infection, cytokine–cytokine receptor interaction, tuberculosis, IL-17 signaling pathway, *Salmonella* infection, viral protein interaction with cytokine and cytokine receptor, Fc gamma R-mediated phagocytosis, human papillomavirus infection, and vibrio cholerae infection.

### The Correlation Between Immune Cells Infiltration and PD-L1 Expression

**Figure 5:**
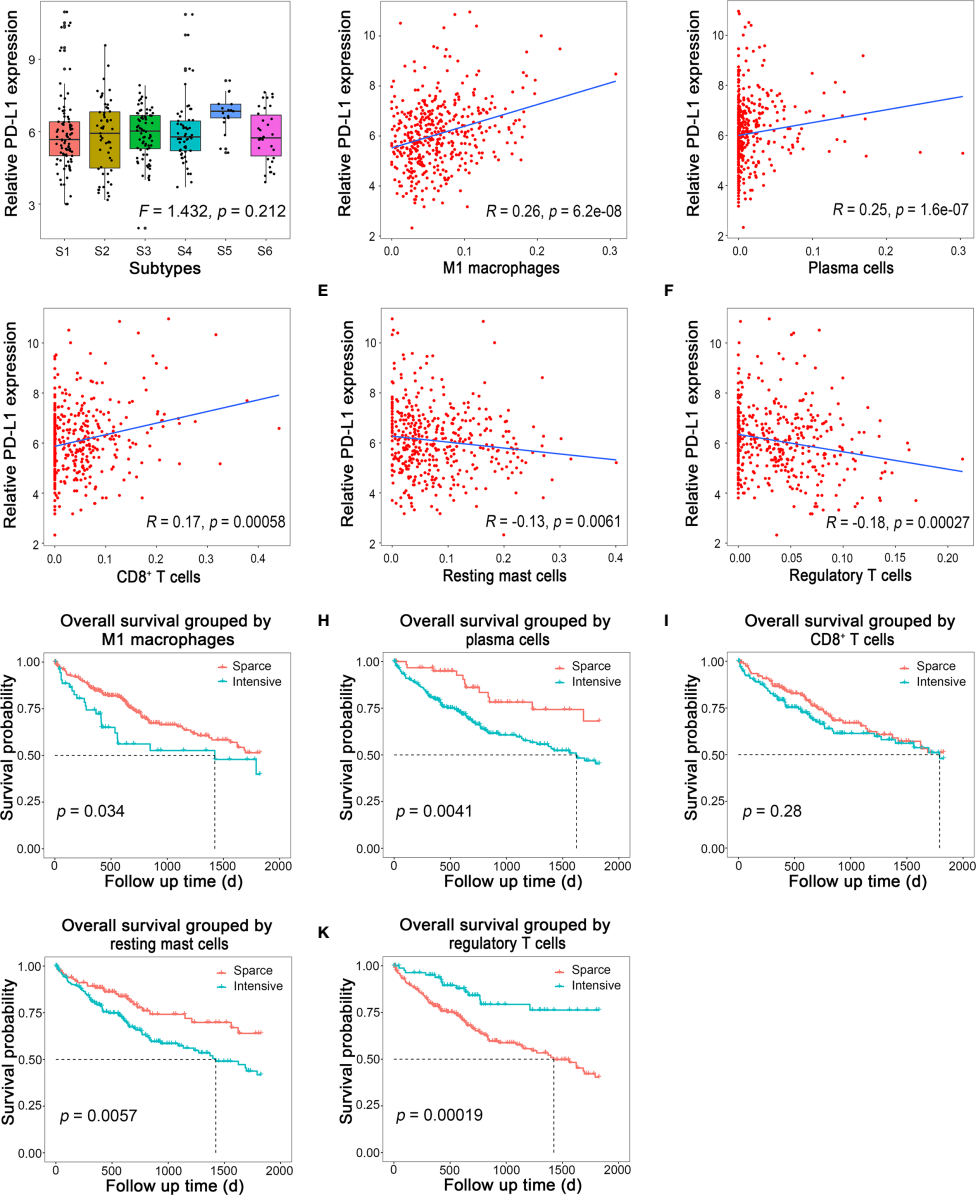
Immune cells related to PD-L1 expression and their prognostic value. **(A)** Relative PD-L1 expression in different immune subtypes in HCC. **(B–F)** Linear regression of PD-L1 and M1 macrophages, plasma cells, CD8+ T cells, resting mast cells, and regulatory T cells, respectively. **(G–K)** Survival curves of M1 macrophages, plasma cells, CD8+ T cells, resting mast cells, and regulatory T cells, respectively.

It has been reported that patients’ responses to anti-PD-1/PD-L1 treatment are positively correlated with the expression level of PD-L1 in tumor tissues (7, 8). Therefore, we would like to figure out whether there was a specific HCC subtype could be characterized by a significantly high PD-L1 level by evaluating the PD-L1 mRNA level among different subtypes. Then, this HCC subtype would be the most potential responders for anti-PD-1/PD-L1 treatment. However, the result showed that even though the PD-L1 mRNA level of S5 ranked the first among all six subtypes, there was no statistical differences among subtypes (**Figure 5A**; *F* = 1.432, *p* = 0.212). In another word, there was no differences in PD-L1 level among different immune subtypes.

Therefore, we suspected that the expression of PD-L1 might be correlated with some specific types of immune cells, other than subtypes of HCC on the basis of immune cell infiltration. To test this hypothesis, the correlation between the fraction of each type of immune cell infiltration and the corresponding PD-L1 mRNA levels was analyzed by Spearman correlation. To our delight, the PD-L1 expression was positively correlated with M1 macrophages (**Figure 5B**; *R* = 0.26, *p* < 0.001), plasma cells (**Figure 5C**; *R* = 0.25, *p* < 0.001), and CD8^+^ T cells (**Figure 5D**; *R* = 0.17, *p* < 0.001), while it was negatively correlated with resting mast cells (**Figure 5E**; *R* = −0.13, *p* = 0.006) and regulatory T cells (**Figure 5F**; *R* = −0.18, *p* < 0.001). The correlation between other immune cells and PD-L1 is shown in **Supplementary Figure S2**. Survival analysis showed that intensive M1 macrophages (**Figure 5G**, *p* = 0.034), plasma cell (**Figure 5H**, *p* = 0.004), and resting mast cells (**Figure 5J**, *p* = 0.006) all indicated poor prognosis, while intensive regulatory T cells (**Figure 5K**, *p* < 0.001) indicated better outcome. The prognosis of HCC was not associated with CD8^+^ T cells (**Figure 5I**, *p* = 0.280).

### Evaluation of PD-L1 Expression and the Infiltration Level of M1 Macrophages and Plasma Cells by Immunohistochemistry of HCC Specimens

**Figure 6:**
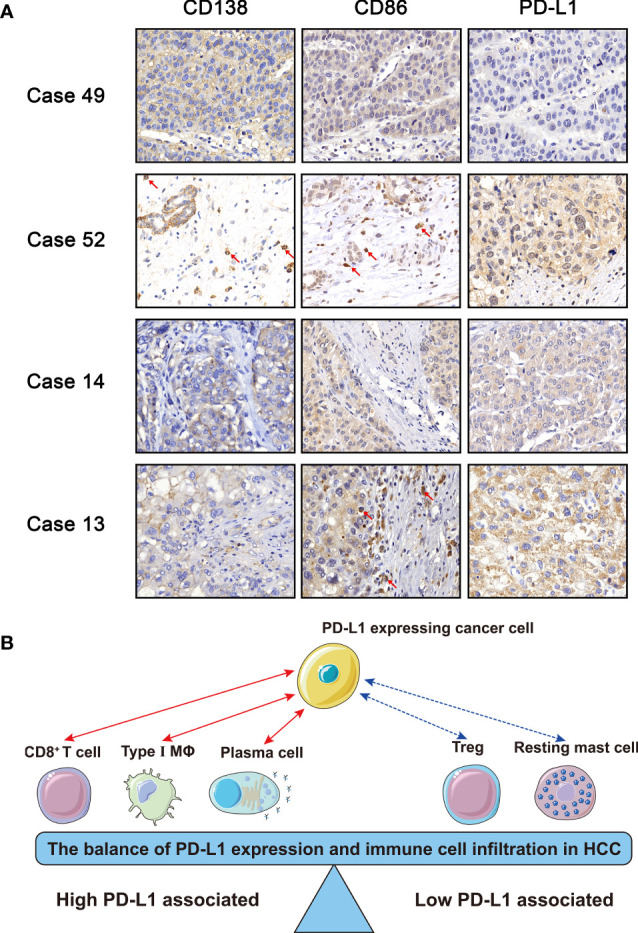
**(A)** Immunohistochemistry of CD138, CD86, and PD-L1. Case 49: − (CD138), − (CD86), and 0 (PD-L1); Case 52: + (CD138), + (CD86), and 2 (PD-L1); Case 14: − (CD138), − (CD86), and 1 (PD-L1); Case 13: − (CD138), + (CD86), and 2 (PD-L1). **(B)** Schematic diagram of the balance of immune cells and PD-L1. The red solid line represented that the PD-L1 expression was positively correlated with M1 macrophages, plasma cells, and CD8^+^ T cells, while the blue dotted line represented that the PD-L1 expression was negatively correlated with resting mast cells and regulatory T cells.

To validate our bioinformatic discovery that the PD-L1 expression level was positively correlated with the infiltration level of plasma cells and M1 macrophages in HCC TME, we performed immunohistochemistry (IHC) analysis on HCC specimens. CD138 is often used as the marker of plasma cells (20), and CD86 is stained to identified M1 macrophage during IHC analysis (21). Representative images of CD138, CD86, and PD-L1 staining were presented, and their staining intensities were quantified (**Figure 6A** and **Supplementary Table S2**). Chi-square test suggested that the expression level of PD-L1 in positive and negative CD86 group was significantly different in HCC specimens (**Table 2**) (*χ^2^* = 5.182, *p* = 0.023), and the odds ratio (OR) was 3.200 [95% confidential interval (95% CI), 1.156, 8.854]. Linear-by-linear association test revealed a positive correlation between CD86 and PD-L1 (*p* = 0.023; Pearson’s *R* = 0.276). As for CD138, its correlation with PD-L1 was not statistically significant, probably due to the relative rarity of plasma cells in TME (*χ^2^* = 1.228, *p* = 0.268). The IHC from our in-house sample demonstrated the bioinformatics finding that the PD-L1 expression was positively correlated with M1 macrophages.

**Table 2:** Chi-square test for evaluation of the relationship of CD86 and PD-L1 based on immunohistochemistry staining.

CD86 expression	PD-L1 expression		χ^2^	p-value
	Low	High		
Negative	28	14	5.182	0.023
Positive	10	16		

### The Correlation Between PD-L1 Expression and M1 Macrophages-Associated Cytokines

Cytokines are produced and excreted by both immune and tumor cells, and they play critical roles in the regulation of immunoreaction in TME. In addition, cytokines can be detected in peripheral blood, which is beneficial for noninvasive examination and real-time dynamic monitoring during cancer treatment. Since we had demonstrated that M1 macrophage infiltration was correlated with high PD-L1 expression in HCC, it appealed to us a lot if there were any correlation between M1 macrophage-associated cytokines and PD-L1 expression.

**Figure 7:**
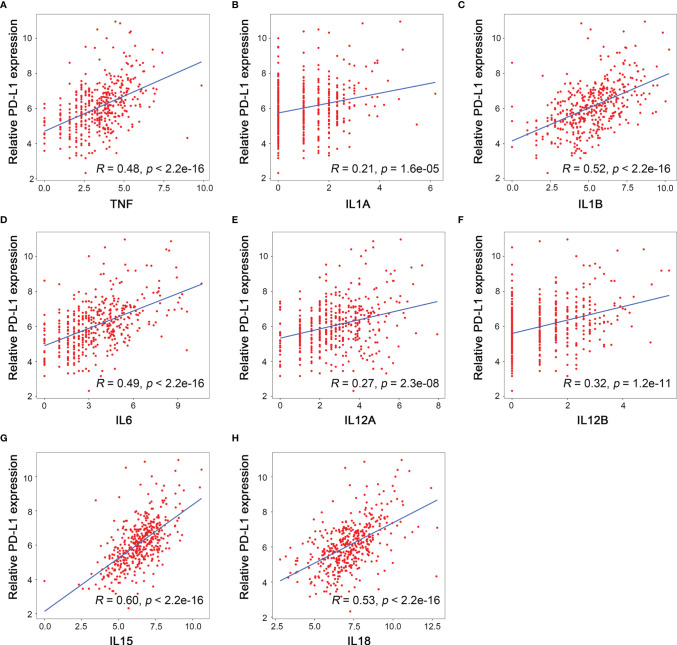
Relationship between the mRNA level of M1 related cytokines and PD-L1 expression. **(A)** TNF, **(B)** IL1A, **(C)** IL1B, **(D)** IL6, **(E)** IL12A, **(F)** IL12B, **(G)** IL15, **(H)** IL18.

M1 macrophages mainly secret proinflammatory, microbicidal, and tumoricidal cytokines such as interleukin (IL)-1, IL-6, IL-12, IL-15, IL-18, and tumor necrosis factor TNF-α (22). The relationship between the mRNA levels of PD-L1 and each of these M1 macrophages-associated cytokines was assessed by linear regression in TCGA-LIHC cohort, and the results suggested that PD-L1 mRNA level was positively correlated with mRNA levels of TNF-α (TNFA, *R* = 0.48, *p* < 0.001), IL-1 (IL1A, *R* = 0.21, *p* < 0.001; IL1B, *R* = 0.52, *p* < 0.001), IL-6 (IL6, *R* = 0.49, *p* < 0.001), IL-12 (IL12A, *R* = 0.27, *p* < 0.001; IL12B, *R* = 0.32, *p* < 0.001), IL-15 (IL15, *R* = 0.60, *p* < 0.001), and IL-18 (IL18, *R* = 0.53, *p* < 0.001) (**Figures 7A–H**). Taken together, PD-L1 expression was positively correlated with not only M1 macrophages but also M1 macrophages-associated cytokines.

## Discussion

Our study found that the status of immune cells infiltration could indicate prognosis and applicability of checkpoint therapy in HCC. HCC patients could be divided into six subtypes according to immune cells infiltration pattern simulated by CIBERSORT algorithm, and their immune microenvironment features indicated different prognosis. The LASSO-logistic model could distinguish HCC and para-cancerous tissues based on immune cells infiltration. In addition, the prognostic model based on the fraction of immune cells was capable to discriminate patients with different prognosis. Although the PD-L1 level did not differ statistically among different immune subtypes, we discovered that there was a linear relationship between PD-L1 level and the fraction of certain immune cells (M1 macrophages, plasma cells, CD8^+^ T cells, resting mast cells, and regulatory T cells). From the perspective of *R*-value, the type of cells showing the strongest relevance with PD-L1 was M1 macrophages, and our immunohistochemistry staining reinforced this conclusion. Furthermore, we found that the mRNA level of M1 macrophage-associated cytokines were positively correlated with the PD-L1 mRNA level, among which the IL-15 showed the strongest correlation.

TME, as the frontline of cancer–host interaction, could be a real-time reflection for patients’ status of anticancer immune reaction. In our present study, the immune cells were categorized into two categories, namely, high and low PD-L1-associated immune cells (**Figure 6B**). The former included CD8^+^ T cells, plasma cells, and M1 macrophages, and the latter included regulatory T cells and resting mast cells.

CD8^+^ T cells are usually referred to as cytotoxic T lymphocytes, which function as the major executers for antitumor immunity. CD8^+^ T cell eliminate malignant cells by perforin, interferon, and TNF. According to our discovery, we speculate that HCC cells might confront CD8^+^ T cells infiltration by upregulating PD-L1 expression. This hypothesis is supported by experimental evidence that CD8^+^ T cells produce cytokines like interferon gamma to induce the upregulation of PD-L1 in melanoma (23). As a consequence of such an adaptive resistance mechanism of immune escape, the prognostic value of CD8^+^ T cell was not as straightforward as its tumoricidal functions. Valerie Chew et al. demonstrated that CD8^+^ T cells were positively correlated with long survival and tumor apoptosis and negatively with tumor proliferation (24), while Thompson et al. showed that higher CD8^+^ T cells infiltration indicated poor prognosis in gastric adenocarcinoma (25). Therefore, CD8^+^ T cells should not be used as a single predictive marker for prognosis or applicability of anti-PD-1/PD-L1 therapy.

In this study, we identified that plasma cell infiltration was also positively correlated with intratumoral PD-L1 expression level in HCC. In early years, researchers considered that antitumor immunity was mainly achieved by cellular immunity mediated by T cells, but recently, more evidence proved that the immunoglobulin (Ig) secreted by B lymphocytes also played an important role in antitumor immunity. It has been discovered that IgG would activate Fc receptors on macrophages and promote M2 polarization and the excretion of IL-6, IL-10, and C-C motif chemokine ligand 20 (CCL20), indicating that plasma cells might exhibit immunosuppressive functions in TME (26, 27). Moreover, intensive plasma cells infiltration was associated with poor prognosis in HCC patients (26, 27). However, the mechanism by which plasma cells infiltration correlates with upregulated PD-L1 expression in HCC cells has not been fully illustrated yet.

Here, we identified that the infiltration of regulatory T cells and resting mast cells was negatively correlated with the PD-L1 expression level in HCC. Regulatory T cells are a well-characterized type of immune cells, which promote the progression of HCC. Several studies have independently demonstrated that the number and the proportion of regulatory T cells increases in HCC compared to surrounding tissue (28). The number of regulatory T cells in peripheral also serves as a biomarker of HCC and disease progression (29). Mechanically, regulatory T cells expressed a variety of immunosuppressive ligands, such as CTLA-4 and PD-1; both are responsible for immunosuppressive in HCC. The recognition of PD-1 on T cells by PD-L1 on regulatory T cells lead to the programmed cell death of T cells so that the immunity is suppressed (30). Currently, Kazushige Yoshida et al. demonstrated tumor-infiltrating regulatory T cells decreased when applying anti-PD-1 antibody (31). However, it is unknown whether regulatory T cells could be the indicator of applicability of anti-PD-1/PD-L1 therapy.

Mast cells are conventionally regarded as the protagonist of anaphylactic reaction, and very few studies have linked mast cells with tumor. The infiltration of mast cells has been reported in colorectal carcinoma (32), cervical carcinoma (33), and breast cancer (34). In HCC, Terada et al. found that mast cells were distributed in the sinusoid and were increased compared to the surrounding liver tissue (35). Although the role of mast cells in cancer remained unclear, the differences in mast cells population between HCC and para-cancerous tissue suggested that mast cells might contribute to disease progression, and the specific functions of mast cells in cancer should be further elucidated in the future.

Generally, our study suggested the existence of a balance between cancer cells and infiltrated immune cells within the immunosuppressive TME (**Figure 6B**). For example, although some HCC patients possess relative high infiltration of CD8^+^ T cells, cancer cells adaptively express PD-L1 to maintain the immunosuppressive TME, which suggests a good response to anti-PD-1/PD-L1 therapy. On the contrary, for patients possessing high proportion of regulatory T cells, the corresponding PD-L1 level of tumor is low. For such patients, anti-PD-1/PD-L1 therapy might not be suitable, while the inhibition of regulatory T cells might work.

In this study, macrophages stand out as the most critical type of infiltrated immune cells in HCC. First, HCC subtypes with high M0 and M2 macrophage infiltration showed worst prognosis. Second, M1 macrophages was strongest correlated with high PD-L1 expression level in HCC. It was widely acknowledged that M1 macrophage possessed antitumor effect, and M2 macrophage contributed to immunosuppression and enhanced HCC progression. In TME, M0 macrophages could either be polarized to M1 macrophages by IFN-γ, TNF-α, or bacterial lipopolysaccharide (LPS) recognition, or be polarized to M2 macrophages by IL-10, IL-4, and IL-13 (36). Since the polarization of macrophages is plastic, our discoveries suggest that macrophage polarization has great potential as immune therapy targets for HCC.

As for M1 macrophages, it could suppress tumor growth by both cytophagocytic effects and the production of TNF-α, reactive oxygen species (ROS), and cytokines like IL-1, IL-12, IL-15, and IL-18 to enhance Th1 response (37). The M1 macrophage-associated cytokines are critical TME components, which should not be ignored. Here, we identified that the correlation between IL-15 and PD-L1 ranked the first among all the cytokines related to M1 macrophages. IL-15 shares similar structure and functions with IL-2, both of which were proinflammatory cytokines and activated “immune-enhancing” signaling cascade (38). Linda Quatrini et al. found that glucocorticoids, together with IL-12, IL-15, and IL-18, induced PD-L1 expression in natural killer cells (39). A phase 1 clinical trial showed that a combination of ALT-803 (an IL-15 superagonist) and nivolumab (monoclonal antibody of PD-1) in non-small cell lung cancer (NSCLC) was safe and showed remarkable response rate, especially in PD-L1-positive patients (40). Haijun Zhou et al. evaluated the prognostic value of IL-15 and IL-2, finding that both of them were independent protective factors for HCC recurrence (41). Emerging evidence has demonstrated that IL-15 might be a promising strategy for cancer immunotherapy (42). Moreover, it was convenient and safe to evaluate the IL-15 level in peripheral blood by commercial enzyme-linked immunosorbent assay (ELISA) kit with a multicytokine/chemokine array. Theresa Vilsmaier et al. demonstrated high IL-15 level in peripheral blood was associated with worse outcome in breast cancer (43). Therefore, our study provides a theoretical basis for noninvasive assessment of intratumoral PD-L1 level by testing IL-15 in peripheral blood.

However, there were still some limitations in the present work. First, the mRNA level of macrophage-associated cytokines could reflect the protein expression of cytokines in a certain extent but not always the same. Therefore, it was imperative to perform cytokine array analysis on samples from HCC patients to validate the findings of this study. Second, there were on stage IV patients in in-house cohort, and it was vague whether our model could be applied to all HCC patients with different stages. Further external validation of our model should be validated. Third, additional clinical or laboratory data of our in-house cohort were missing in this study, and it was imperative to perform further analysis to reveal the relationship between different clinical characteristics and our finding. Lastly, quantified methods such as fluorescence-activated cell sorting (FACS) should be further applied to validate the bioinformatics and IHC conclusion that macrophage was positively correlated to PD-L1 level. Recently, Jianpeng Sheng et al. performed topological analysis based on imaging mass cytometry to reveal the microenvironment in HCC (44) and shedding some light on the realm of immune–cancer interaction in HCC.

## Conclusion

Overall, we grouped HCC patients based on the landscape of immune cells, which was feasible to predict the prognosis of HCC patients. Moreover, we also analyzed the relationship between PD-L1, immune cells, and M1 macrophage-related cytokines, which was instructive for noninvasive screening of the applicability of anti PD-1/PD-L1 treatment in the future.

## Data Availability Statement

The original contributions presented in the study are included in the article/**Supplementary Material**. Further inquiries can be directed to the corresponding authors.

## Ethics Statement

The studies involving human participants were reviewed and approved by National Cancer Centre Singapore and The First Affiliated Hospital, Zhejiang University School of Medicine. The patients/participants provided their written informed consent to participate in this study.

## Author Contributions

YQ and SZ conceived the idea, designed the study, and supervised our work. JH and LeZ performed statistical analysis, bioinformatics analysis, immunohistochemistry staining, and drafted the manuscript. JC assisted on information collection of Singapore cohort and revised our manuscript. DW and LiZ helped revise our manuscript. All authors contributed to the article and approved the submitted version.

## Funding

This study is supported by the National Natural Science Foundation of China (No. 81903143 to YQ), Innovative Research Groups of National Natural Science Foundation of China (No. 81721091 to SZ), and National S&T Major Project (No. 2017ZX10203205 to SZ).

## Conflict of Interest

The authors declare that the research was conducted in the absence of any commercial or financial relationships that could be construed as a potential conflict of interest.

## Publisher’s Note

All claims expressed in this article are solely those of the authors and do not necessarily represent those of their affiliated organizations, or those of the publisher, the editors and the reviewers. Any product that may be evaluated in this article, or claim that may be made by its manufacturer, is not guaranteed or endorsed by the publisher.

## Supplementary Material

Supplementary Figure 1 Hematoxylin-eosin (H&E) staining revealed that there were areas highly infiltrated with immune cells and poorly infiltrated areas.

Supplementary Figure 2 The relationship between immune cells and PD-L1 expression. **(A)** Naïve B cells. **(B)** Memory B cells. **(C)** Naïve CD4+ T cells. **(D)** Resting CD4+ memory T cells. **(E)** Activated CD4+ memory T cells. **(F)** T follicular helper cells. **(G)** Gamma delta T cells. **(H)** Resting NK cells. **(I)** Activated NK cells. **(J)** Monocytes. **(K)** M0 macrophages. **(L)** M2 macrophages. **(M)** Resting dendritic cells. **(N)** Activated dendritic cells. **(O)** Activated mast cells. **(P)** Eosinophils. **(Q)** Neutrophils.

Supplementary Table 1 Potential pathways related to high and low pIRS score.

Supplementary Table 2 Immunochemistry evaluation of CD138, CD86, and PD-L1.

## Abbreviations

HCC, hepatocellular carcinoma; PD-1, programmed cell death protein 1; PD-L1, programmed death ligand 1; CTLA-4, cytotoxic T-lymphocyte-associated protein 4; CAR-T cell, chimeric antigen receptor T cell; VEGF, vascular endothelial growth factor; TME, tumor microenvironment; iNKT, invariant natural killer T cells; H&E, hematoxylin–eosin; CIBERSORT, Cell-Type Identification by Estimating Relative Subsets of RNA Transcripts; TCGA, The Cancer Genome Atlas; LIHC, liver hepatocellular carcinoma; HTSeq counts, high-throughput sequencing counts; LASSO, least absolute shrinkage and selection operator; dIRS, diagnostic immune risk score; ROC curves, receiver operating characteristic curves; OS, overall survival; pIRS, prognostic immune risk score; AUC, area under the curve; ANOVA, analysis of variance; GSEA, gene set enrichment analysis; DAB, diaminobenzidine; KEGG, Kyoto Encyclopedia of Genes and Genomes; IHC, immunohistochemistry; OR, odds ratio; 95% CI, 95% confidential interval; IL, interleukin; TNF, tumor necrosis factor; Ig, immunoglobulin; CCL20, C–C motif chemokine ligand 20; LPS, lipopolysaccharide; ROS, reactive oxygen species; NSCLC, non-small cell lung cancer; ELISA, enzyme-linked immunosorbent assay; FACS, Fluorescence Activated Cell Sorting.
