# Supplementary figures and images for: The Landscape of Immune Cells Indicates Prognosis and Applicability of Checkpoint Therapy in Hepatocellular Carcinoma
Source: Front Oncol. 2021 Sep 28;11:744951. doi: 10.3389/fonc.2021.744951 (PMC8510566; doi:10.3389/fonc.2021.744951)

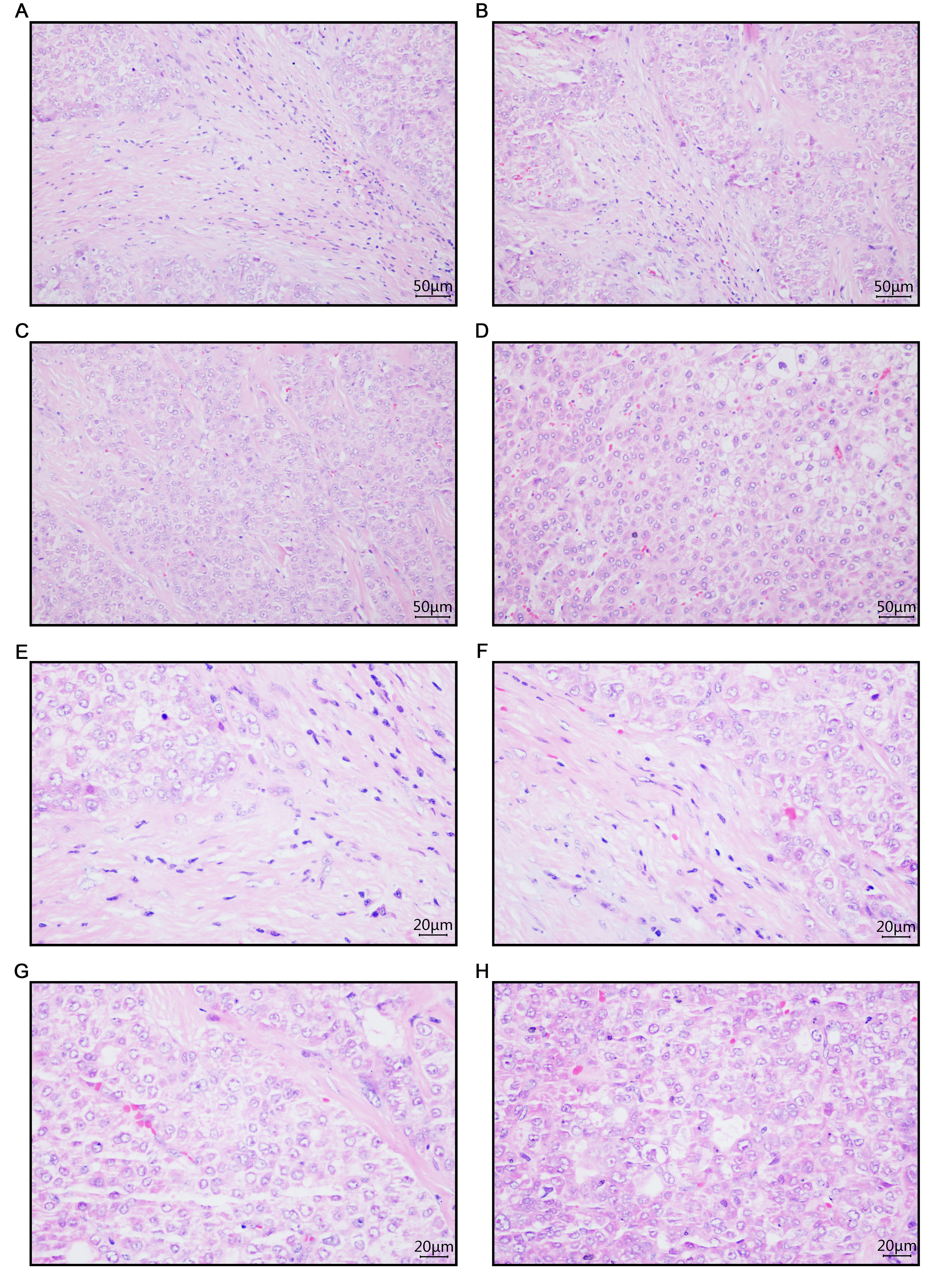

Supplement: Supplementary Figure 1 — Hematoxylin-eosin (H&E) staining revealed that there were areas highly infiltrated with immune cells and poorly infiltrated areas. [file Image_1.tif]

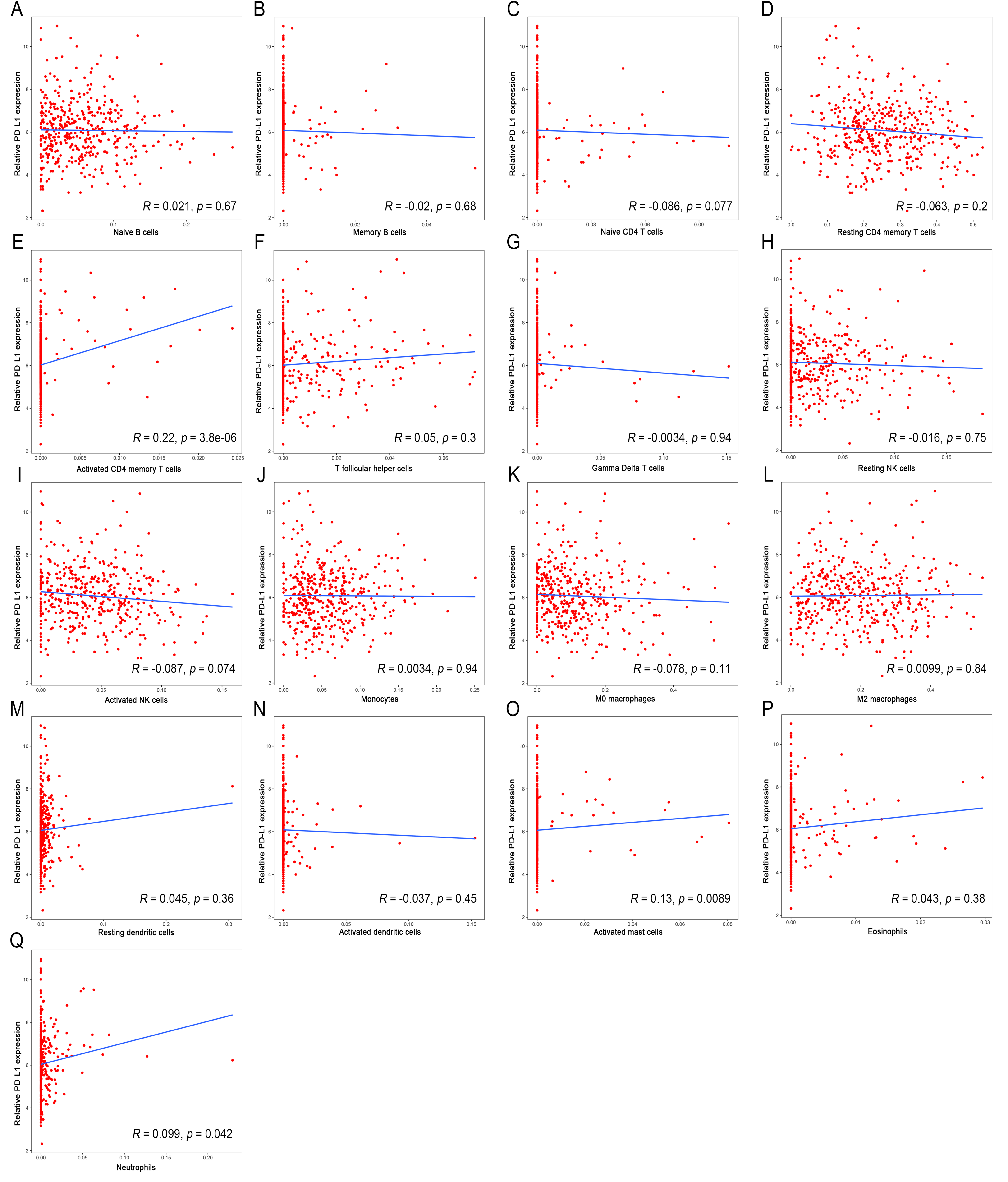

Supplement: Supplementary Figure 2 — The relationship between immune cells and PD-L1 expression. (A) Naïve B cells. (B) Memory B cells. (C) Naïve CD4+ T cells. (D) Resting CD4+ memory T cells. (E) Activated CD4+ memory T cells. (F) T follicular helper cells. (G) Gamma delta T cells. (H) Resting NK cells. (I) Activated NK cells. (J) Monocytes. (K) M0 macrophages. (L) M2 macrophages. (M) Resting dendritic cells. (N) Activated dendritic cells. (O) Activated mast cells. (P) Eosinophils. (Q) Neutrophils. [file Image_2.tif]
