# Supplementary material for: The Landscape of Immune Cells Indicates Prognosis and Applicability of Checkpoint Therapy in Hepatocellular Carcinoma
Source: Front Oncol. 2021 Sep 28;11:744951. doi: 10.3389/fonc.2021.744951 (PMC8510566; doi:10.3389/fonc.2021.744951)
Supplement: Supplementary Table 1 — Potential pathways related to high and low pIRS score. [file Table_1.docx]

Supplementary Table 1. Potential pathways related to high and low pIRS score.

| ID | Description | setSize | enrichmentScore | NES | pvalue | p.adjust | qvalues | rank |
| --- | --- | --- | --- | --- | --- | --- | --- | --- |
| hsa00071 | Fatty acid degradation | 44 | 0.706937 | 3.150641 | 1.00E-10 | 5.37E-09 | 3.56E-09 | 1071 |
| hsa00830 | Retinol metabolism | 66 | 0.57026 | 2.734907 | 1.00E-10 | 5.37E-09 | 3.56E-09 | 1184 |
| hsa00980 | Metabolism of xenobiotics by cytochrome P450 | 71 | 0.570935 | 2.78385 | 1.00E-10 | 5.37E-09 | 3.56E-09 | 1184 |
| hsa00982 | Drug metabolism - cytochrome P450 | 66 | 0.630114 | 3.021963 | 1.00E-10 | 5.37E-09 | 3.56E-09 | 1251 |
| hsa04610 | Complement and coagulation cascades | 85 | 0.52896 | 2.706476 | 1.00E-10 | 5.37E-09 | 3.56E-09 | 1434 |
| hsa05204 | Chemical carcinogenesis | 79 | 0.585386 | 2.862305 | 1.00E-10 | 5.37E-09 | 3.56E-09 | 1756 |
| hsa00260 | Glycine, serine and threonine metabolism | 40 | 0.668741 | 3.000683 | 5.30E-10 | 2.44E-08 | 1.62E-08 | 1001 |
| hsa03320 | PPAR signaling pathway | 76 | 0.489718 | 2.408694 | 2.62E-09 | 1.06E-07 | 7.01E-08 | 1397 |
| hsa00140 | Steroid hormone biosynthesis | 60 | 0.5326 | 2.571958 | 1.44E-08 | 4.85E-07 | 3.22E-07 | 1184 |
| hsa04146 | Peroxisome | 82 | 0.456543 | 2.259253 | 1.50E-08 | 4.85E-07 | 3.22E-07 | 1077 |
| hsa00220 | Arginine biosynthesis | 21 | 0.790382 | 3.080636 | 1.92E-08 | 5.61E-07 | 3.73E-07 | 760 |
| hsa00120 | Primary bile acid biosynthesis | 17 | 0.823582 | 3.027736 | 3.88E-08 | 1.04E-06 | 6.92E-07 | 1455 |
| hsa04976 | Bile secretion | 89 | 0.426135 | 2.203735 | 8.58E-08 | 2.12E-06 | 1.41E-06 | 1422 |
| hsa00350 | Tyrosine metabolism | 35 | 0.62502 | 2.707225 | 3.52E-07 | 8.09E-06 | 5.37E-06 | 940 |
| hsa05323 | Rheumatoid arthritis | 89 | -0.55723 | -1.84665 | 4.52E-07 | 9.69E-06 | 6.43E-06 | 5501 |
| hsa04614 | Renin-angiotensin system | 23 | 0.693979 | 2.732742 | 9.81E-07 | 1.97E-05 | 1.31E-05 | 282 |
| hsa04145 | Phagosome | 148 | -0.4934 | -1.6939 | 1.68E-06 | 3.19E-05 | 2.12E-05 | 4903 |
| hsa00983 | Drug metabolism - other enzymes | 77 | 0.397735 | 1.965068 | 3.63E-06 | 6.50E-05 | 4.31E-05 | 1184 |
| hsa05219 | Bladder cancer | 41 | -0.63974 | -1.95549 | 4.37E-06 | 7.40E-05 | 4.91E-05 | 3215 |
| hsa05150 | Staphylococcus aureus infection | 91 | -0.53471 | -1.7759 | 5.80E-06 | 9.34E-05 | 6.20E-05 | 2751 |
| hsa00340 | Histidine metabolism | 22 | 0.65368 | 2.553092 | 1.35E-05 | 0.000206 | 0.000137 | 1071 |
| hsa04060 | Cytokine-cytokine receptor interaction | 292 | -0.41296 | -1.45676 | 1.82E-05 | 0.000266 | 0.000177 | 2755 |
| hsa05140 | Leishmaniasis | 73 | -0.54888 | -1.78569 | 2.00E-05 | 0.00028 | 0.000186 | 5627 |
| hsa04979 | Cholesterol metabolism | 50 | 0.455747 | 2.149533 | 2.51E-05 | 0.000337 | 0.000224 | 1167 |
| hsa04110 | Cell cycle | 124 | -0.48126 | -1.63729 | 3.60E-05 | 0.000457 | 0.000303 | 3929 |
| hsa05130 | Pathogenic Escherichia coli infection | 191 | -0.44675 | -1.54567 | 3.69E-05 | 0.000457 | 0.000303 | 4014 |
| hsa00380 | Tryptophan metabolism | 42 | 0.475643 | 2.10742 | 6.41E-05 | 0.000753 | 0.0005 | 1899 |
| hsa04512 | ECM-receptor interaction | 88 | -0.50714 | -1.67859 | 6.55E-05 | 0.000753 | 0.0005 | 2277 |
| hsa00270 | Cysteine and methionine metabolism | 49 | 0.429972 | 2.01801 | 0.000139 | 0.001547 | 0.001027 | 760 |
| hsa05152 | Tuberculosis | 176 | -0.43669 | -1.50785 | 0.000161 | 0.001725 | 0.001145 | 6118 |
| hsa04380 | Osteoclast differentiation | 125 | -0.46555 | -1.58523 | 0.000193 | 0.001966 | 0.001305 | 3294 |
| hsa04810 | Regulation of actin cytoskeleton | 214 | -0.42076 | -1.46436 | 0.000195 | 0.001966 | 0.001305 | 5570 |
| hsa04657 | IL-17 signaling pathway | 94 | -0.48995 | -1.63231 | 0.000221 | 0.002156 | 0.001431 | 3116 |
| hsa00630 | Glyoxylate and dicarboxylate metabolism | 30 | 0.538511 | 2.265175 | 0.000241 | 0.002287 | 0.001518 | 1157 |
| hsa00910 | Nitrogen metabolism | 17 | 0.635794 | 2.337371 | 0.000265 | 0.002442 | 0.00162 | 710 |
| hsa00601 | Glycosphingolipid biosynthesis - lacto and neolacto series | 27 | -0.64408 | -1.84047 | 0.000307 | 0.00275 | 0.001825 | 2453 |
| hsa04390 | Hippo signaling pathway | 156 | -0.43966 | -1.5144 | 0.000351 | 0.003054 | 0.002027 | 3431 |
| hsa04142 | Lysosome | 128 | -0.4451 | -1.51671 | 0.000383 | 0.003249 | 0.002156 | 6939 |
| hsa05410 | Hypertrophic cardiomyopathy | 90 | -0.49084 | -1.62732 | 0.000472 | 0.003894 | 0.002584 | 2439 |
| hsa00010 | Glycolysis / Gluconeogenesis | 67 | 0.352226 | 1.694296 | 0.000552 | 0.004441 | 0.002947 | 596 |
| hsa05206 | MicroRNAs in cancer | 161 | -0.42938 | -1.48078 | 0.000696 | 0.005465 | 0.003627 | 3145 |
| hsa05133 | Pertussis | 76 | -0.4961 | -1.61813 | 0.000736 | 0.005639 | 0.003742 | 2672 |
| hsa01230 | Biosynthesis of amino acids | 74 | 0.345119 | 1.70908 | 0.000785 | 0.005876 | 0.003899 | 760 |
| hsa04144 | Endocytosis | 247 | -0.39457 | -1.38207 | 0.001042 | 0.00759 | 0.005037 | 8741 |
| hsa05132 | Salmonella infection | 213 | -0.40698 | -1.41622 | 0.001061 | 0.00759 | 0.005037 | 8485 |
| hsa00280 | Valine, leucine and isoleucine degradation | 48 | 0.387581 | 1.797632 | 0.001166 | 0.008163 | 0.005417 | 2574 |
| hsa04061 | Viral protein interaction with cytokine and cytokine receptor | 98 | -0.45778 | -1.53067 | 0.001199 | 0.008215 | 0.005452 | 2535 |
| hsa04666 | Fc gamma R-mediated phagocytosis | 92 | -0.46685 | -1.55033 | 0.001267 | 0.008339 | 0.005534 | 6162 |
| hsa04975 | Fat digestion and absorption | 42 | 0.405693 | 1.797495 | 0.001269 | 0.008339 | 0.005534 | 1865 |
| hsa05134 | Legionellosis | 57 | -0.51384 | -1.64112 | 0.001382 | 0.008898 | 0.005905 | 3571 |
| hsa00650 | Butanoate metabolism | 28 | 0.480799 | 1.952532 | 0.00151 | 0.009532 | 0.006325 | 846 |
| hsa00360 | Phenylalanine metabolism | 16 | 0.568966 | 2.043214 | 0.001644 | 0.010182 | 0.006757 | 940 |
| hsa05016 | Huntington disease | 292 | -0.376 | -1.3264 | 0.001962 | 0.011722 | 0.007779 | 7496 |
| hsa05131 | Shigellosis | 239 | -0.38456 | -1.34697 | 0.001999 | 0.011722 | 0.007779 | 6091 |
| hsa05120 | Epithelial cell signaling in Helicobacter pylori infection | 70 | -0.48857 | -1.5874 | 0.002019 | 0.011722 | 0.007779 | 6331 |
| hsa04920 | Adipocytokine signaling pathway | 69 | 0.33527 | 1.602102 | 0.002039 | 0.011722 | 0.007779 | 1579 |
| hsa04931 | Insulin resistance | 108 | 0.275666 | 1.429877 | 0.002188 | 0.012254 | 0.008132 | 1579 |
| hsa04514 | Cell adhesion molecules | 145 | -0.41712 | -1.4305 | 0.002245 | 0.012254 | 0.008132 | 4794 |
| hsa05165 | Human papillomavirus infection | 331 | -0.36982 | -1.30944 | 0.002245 | 0.012254 | 0.008132 | 6247 |
| hsa05205 | Proteoglycans in cancer | 202 | -0.39349 | -1.3659 | 0.002373 | 0.012526 | 0.008312 | 4542 |
| hsa00591 | Linoleic acid metabolism | 29 | 0.457911 | 1.908683 | 0.002373 | 0.012526 | 0.008312 | 1865 |
| hsa00250 | Alanine, aspartate and glutamate metabolism | 36 | 0.425074 | 1.852354 | 0.002424 | 0.012587 | 0.008353 | 846 |
| hsa01200 | Carbon metabolism | 117 | 0.266999 | 1.460748 | 0.002486 | 0.012708 | 0.008433 | 1220 |
| hsa04922 | Glucagon signaling pathway | 106 | 0.286318 | 1.485964 | 0.002857 | 0.014373 | 0.009538 | 1579 |
| hsa00040 | Pentose and glucuronate interconversions | 34 | 0.409378 | 1.79445 | 0.004872 | 0.024133 | 0.016015 | 1184 |
| hsa04530 | Tight junction | 161 | -0.40504 | -1.39683 | 0.005271 | 0.025716 | 0.017066 | 4399 |
| hsa05014 | Amyotrophic lateral sclerosis | 349 | -0.35899 | -1.27391 | 0.005445 | 0.026168 | 0.017365 | 7502 |
| hsa05414 | Dilated cardiomyopathy | 95 | -0.44215 | -1.4726 | 0.005595 | 0.026494 | 0.017582 | 2439 |
| hsa00053 | Ascorbate and aldarate metabolism | 27 | 0.442677 | 1.805525 | 0.005739 | 0.02678 | 0.017772 | 1184 |
| hsa05230 | Central carbon metabolism in cancer | 69 | -0.46604 | -1.51366 | 0.005935 | 0.027042 | 0.017946 | 2931 |
| hsa04923 | Regulation of lipolysis in adipocytes | 55 | 0.330859 | 1.605339 | 0.005963 | 0.027042 | 0.017946 | 1330 |
| hsa05110 | Vibrio cholerae infection | 50 | -0.49704 | -1.56064 | 0.006518 | 0.02915 | 0.019345 | 5993 |
| hsa05217 | Basal cell carcinoma | 63 | -0.48092 | -1.54901 | 0.006701 | 0.029557 | 0.019615 | 2394 |
| hsa03010 | Ribosome | 134 | -0.40847 | -1.39727 | 0.006854 | 0.029824 | 0.019792 | 9655 |
| hsa04392 | Hippo signaling pathway - multiple species | 28 | -0.54907 | -1.57983 | 0.006991 | 0.030013 | 0.019917 | 2990 |
| hsa00565 | Ether lipid metabolism | 49 | -0.49454 | -1.55119 | 0.007998 | 0.033888 | 0.022488 | 3960 |
| hsa04510 | Focal adhesion | 201 | -0.38062 | -1.3203 | 0.00846 | 0.035177 | 0.023344 | 3670 |
| hsa00603 | Glycosphingolipid biosynthesis - globo and isoglobo series | 15 | -0.63893 | -1.60554 | 0.008521 | 0.035177 | 0.023344 | 2714 |
| hsa05146 | Amoebiasis | 101 | -0.42378 | -1.42175 | 0.010851 | 0.043685 | 0.02899 | 4025 |
| hsa00410 | beta-Alanine metabolism | 30 | 0.429547 | 1.806831 | 0.010853 | 0.043685 | 0.02899 | 1071 |
| hsa05010 | Alzheimer disease | 354 | -0.35024 | -1.2432 | 0.011547 | 0.045904 | 0.030463 | 7534 |
| hsa04360 | Axon guidance | 181 | -0.3726 | -1.28692 | 0.011814 | 0.04639 | 0.030785 | 6214 |
| hsa00052 | Galactose metabolism | 31 | -0.52945 | -1.54372 | 0.012132 | 0.047065 | 0.031233 | 2554 |
| hsa00533 | Glycosaminoglycan biosynthesis - keratan sulfate | 14 | -0.63568 | -1.57337 | 0.012558 | 0.048138 | 0.031945 | 3723 |
| hsa04978 | Mineral absorption | 59 | -0.46467 | -1.49012 | 0.013101 | 0.049172 | 0.032632 | 2965 |
| hsa00330 | Arginine and proline metabolism | 49 | 0.328717 | 1.542782 | 0.013133 | 0.049172 | 0.032632 | 1071 |
