# Supplementary material for: The Landscape of Immune Cells Indicates Prognosis and Applicability of Checkpoint Therapy in Hepatocellular Carcinoma
Source: Front Oncol. 2021 Sep 28;11:744951. doi: 10.3389/fonc.2021.744951 (PMC8510566; doi:10.3389/fonc.2021.744951)
Supplement: Supplementary Table 2 — Immunochemistry evaluation of CD138, CD86, and PD-L1. [file Table_2.docx]

Supplementary Table 2. Immunochemistry evaluation of CD138, CD86, and PD-L1.

| Patients ID | CD138 | CD86 | Final  PD-L1 | Reviewer 1  PD-L1 | Reviewer 2  PD-L1 | Reviewer 3  PD-L1 |
| --- | --- | --- | --- | --- | --- | --- |
| 1 | + | + | 1 | 1 | 1 | 1 |
| 2 | - | - | 2 | 2 | 2 | 2 |
| 3 | - | - | 2 | 2 | 2 | 2 |
| 4 | - | + | 0.333333 | 0 | 1 | 0 |
| 5 | - | + | 2 | 2 | 2 | 2 |
| 6 | - | + | 1 | 1 | 1 | 1 |
| 7 | + | + | 2 | 2 | 2 | 2 |
| 8 | - | + | 2 | 2 | 2 | 2 |
| 9 | - | + | 3 | 3 | 3 | 3 |
| 10 | - | + | 1.666667 | 1 | 2 | 2 |
| 11 | + | + | 2 | 2 | 2 | 2 |
| 12 | + | + | 1.333333 | 1 | 2 | 1 |
| 13 | - | + | 2 | 2 | 2 | 2 |
| 14 | - | - | 1 | 1 | 1 | 1 |
| 15 | - | + | 1 | 1 | 1 | 1 |
| 16 | - | - | 0 | 0 | 0 | 0 |
| 17 | - | + | 1 | 1 | 1 | 1 |
| 18 | - | - | 1.333333 | 1 | 2 | 1 |
| 19 | - | - | 0.666667 | 0 | 1 | 1 |
| 20 | - | - | 1.333333 | 1 | 2 | 1 |
| 21 | + | - | 0.666667 | 0 | 1 | 1 |
| 22 | - | - | 1.333333 | 1 | 2 | 1 |
| 23 | + | - | 1.333333 | 1 | 2 | 1 |
| 24 | + | + | 0.666667 | 0 | 1 | 1 |
| 25 | - | - | 0.333333 | 0 | 1 | 0 |
| 26 | - | - | 1.333333 | 1 | 2 | 1 |
| 27 | - | - | 0.333333 | 0 | 1 | 0 |
| 28 | + | - | 0 | 0 | 0 | 0 |
| 29 | - | - | 1 | 1 | 1 | 1 |
| 30 | - | - | 1 | 1 | 1 | 1 |
| 31 | + | - | 0.666667 | 1 | 1 | 0 |
| 32 | + | - | 0 | 0 | 0 | 0 |
| 33 | + | + | 0.333333 | 0 | 1 | 0 |
| 34 | - | - | 1 | 1 | 1 | 1 |
| 35 | - | - | 0 | 0 | 0 | 0 |
| 36 | - | + | 1.333333 | 1 | 2 | 1 |
| 37 | + | + | 2 | 2 | 2 | 2 |
| 38 | + | - | 0.666667 | 0 | 1 | 1 |
| 39 | + | - | 0 | 0 | 0 | 0 |
| 40 | + | - | 1 | 1 | 1 | 1 |
| 41 | + | - | 0.333333 | 0 | 1 | 0 |
| 42 | - | - | 2.333333 | 2 | 3 | 2 |
| 43 | - | - | 1.333333 | 1 | 2 | 1 |
| 44 | - | + | 0.666667 | 1 | 1 | 0 |
| 45 | + | - | 0 | 0 | 0 | 0 |
| 46 | - | + | 1.666667 | 1 | 2 | 2 |
| 47 | + | - | 0.666667 | 1 | 1 | 0 |
| 48 | - | - | 1 | 1 | 1 | 1 |
| 49 | - | - | 0 | 0 | 0 | 0 |
| 50 | - | - | 1 | 1 | 1 | 1 |
| 51 | - | - | 1 | 1 | 1 | 1 |
| 52 | + | + | 2 | 2 | 2 | 2 |
| 53 | - | - | 1.333333 | 1 | 2 | 1 |
| 54 | - | - | 1 | 1 | 1 | 1 |
| 55 | - | + | 1.666667 | 1 | 2 | 2 |
| 56 | - | - | 1 | 1 | 1 | 1 |
| 57 | + | + | 1.333333 | 1 | 1 | 2 |
| 58 | - | - | 0.333333 | 0 | 1 | 0 |
| 59 | + | - | 0.666667 | 1 | 1 | 0 |
| 60 | + | + | 2 | 2 | 2 | 2 |
| 61 | - | - | 1.333333 | 1 | 2 | 1 |
| 62 | - | - | 2.333333 | 2 | 3 | 2 |
| 63 | - | + | 0.333333 | 0 | 1 | 0 |
| 64 | - | - | 1 | 1 | 1 | 1 |
| 65 | - | + | 1 | 1 | 1 | 1 |
| 66 | - | + | 0.333333 | 0 | 0 | 1 |
| 67 | - | - | 0.666667 | 1 | 1 | 0 |
| 68 | + | - | 0.333333 | 0 | 1 | 0 |
